# Idiopathic Mediastinal Fibrosis: A Study of a Case Using CT and Bronchoscopic Imaging

**DOI:** 10.7759/cureus.55344

**Published:** 2024-03-01

**Authors:** Khadija Chaanoun, Fatima Ezzahra Haouassia, Nahid Zaghba, Hanane Benjelloun, Najiba Yassine

**Affiliations:** 1 Pulmonology Department, Ibn Rochd University Hospital, Casablanca, MAR; 2 Pulmonology Department, CHU (Centre Hospitalier Universitaire) Ibn Rochd, Casablanca, MAR

**Keywords:** corticosteroid medication, chronic obstructive broncho-pneumopathy, mediastinoscopy, cancer, fibrosing mediastinitis

## Abstract

An uncommon illness known as fibrosing mediastinitis causes the mediastinum to grow excessively thick fibrous tissue. Fungal or idiopathic origins are the most common etiologies of pathology. In an individual suffering from chronic obstructive pulmonary disease (COPD), fibrosing mediastinitis, which resembled a bronchogenic cancer, was identified during anatomopathological examination following mediastinoscopy.

## Introduction

Mediastinal fibrosis, also known as fibrous mediastinitis, occurs when collagen fibers accumulate over time in the mediastinum's connective tissue, putting pressure on the organs and eventually spreading to them. This phenomenon is uncommon [1]; only a few instances are documented in the literature. Most cases are classified as idiopathic. Secondary forms, resulting from a previous infection, have occurred.

## Case presentation

Mr. A.H., 52, a habitual smoker of 30 packs per year with no other specific pathological history, developed grade 2 mMRC (Modified Medical Research Council) dyspnea, a dry cough, and purulent sputum as part of his Stage B chronic obstructive pulmonary disease (COPD).

The clinical examination at admission showed a blood pressure of 130/70 mmHg and a body mass index of 22 kg/m^2^. The heart rate was 82 beats per minute, and the blood oxygen saturation was 95% when exposed to normal air conditions. The pulmonary auscultation indicated the presence of bilateral sonorous rales. There was an absence of lymph node enlargement or swelling in the lower extremities.

Blood investigation showed that the complete blood count (CBC) was normal; the leukocytes level was 8,900/mm^3^, and 58% of the cells were neutrophils and polynuclear. The C-reactive protein (CRP) level was 21 milligrams per liter (mg/L), and the fibrinogen level was 6.11 grams per liter (g/L). The levels of ions, renal function, and liver tests were within the normal range.

**Figure 1:**
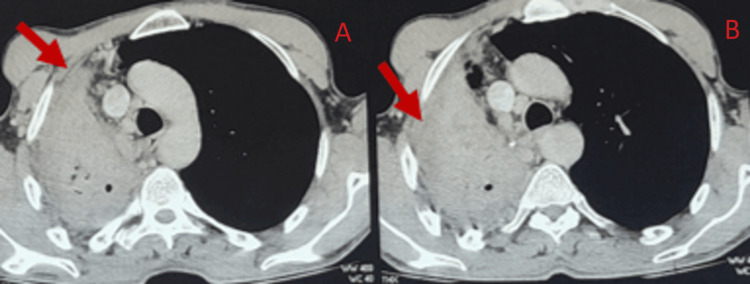
Lobular mediastinal mass that looks like tissue and includes parts of the mediastinum.

A chest CT scan showed a lobular mediastinal mass that looked like tissue and included parts of the mediastinum (Figure 1).

**Figure 2:**
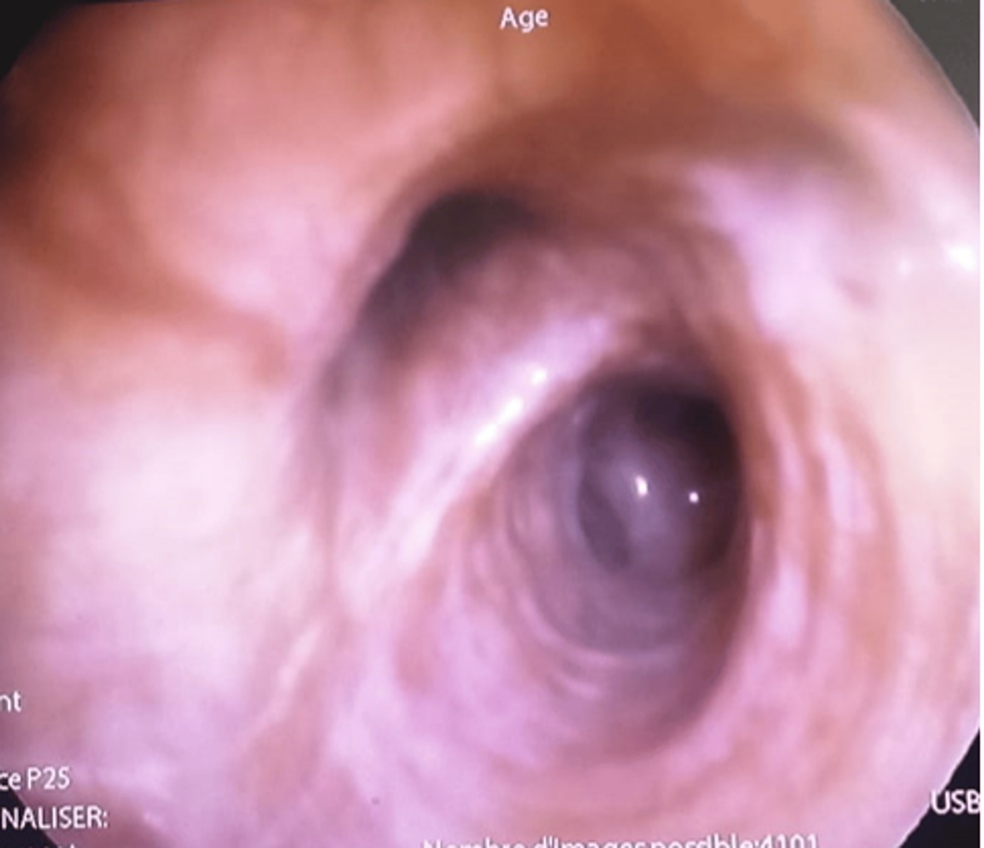
The flexible bronchoscopy revealed an external compression condition in addition to a moderate inflammatory condition.

The flexible bronchoscopy revealed an external compression condition in addition to a moderate inflammatory condition (Figure 2).

The histopathological examination of bronchial samples revealed fibro-inflammatory remodeling that was not specific.

In the discipline of oncology-thoracic oncology, the patient's harmful habits resulted in the suspicion of a bronchogenic carcinoma, which necessitated the choice to conduct a mediastinoscopy. The latter was executed. It revealed a hard, fibrous mass. An anatomopathological study of the mediastinal mass revealed mediastinal fibrosis. The etiological survey yielded negative results, indicating the absence of similar cases within the family and ruling out any history of medicine or radiation therapy. We have successfully excluded tuberculosis, sarcoidosis, and silicosis based on anatomopathological analysis. The microbiological analysis of the biopsy fragments yielded negative results. The results of the immunological and thyroid tests were negative.

The abdominal ultrasound revealed no anomalies. We acknowledged the diagnosis of idiopathic fibrous mediastinitis.

**Figure 3:**
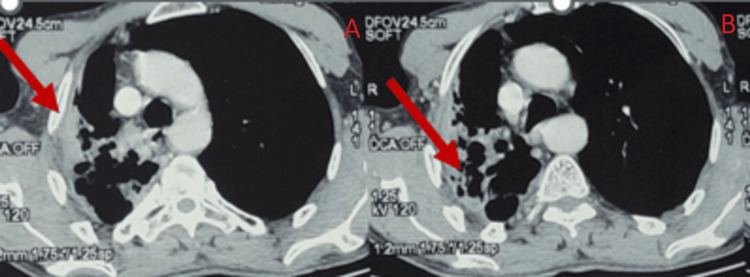
Apparent air-filled bronchial tubes and aberrant bronchial tube dilatation in the form of a cystic and cylindrical structure, together with regional collapse of the apical and dorsal portions of the right lower lung lobe. The image was obtained using the mediastinal window of the thoracic scanner.

**Figure 4:**
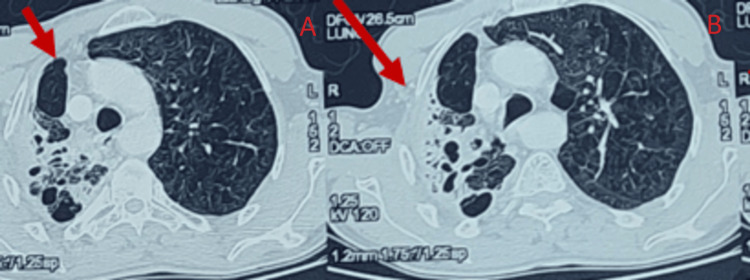
Localized collapse of the apical and dorsal segments of the right lower lung lobe, with visible air-filled bronchial tubes and abnormal dilatation of the bronchial tubes in a cystic and cylindrical shape. The image was taken using the parenchymal window of the chest scanner.

A decision was made to reassess the chest CT scan before initiating prolonged corticosteroid treatment. The thoracic scan of the control subject showed a difference in volume between the two pulmonary hemispheres. The examination also detected a localized collapse of the apical and dorsal segments of the left lower lung lobe, with visible air-filled bronchial tubes and abnormal dilatation of the bronchial tubes in a cystic and cylindrical shape. The examination ended with the observation of a harmonious appearance of a non-obstructive tissue sleeve surrounding the right stem bronchus (Figures 3, 4).

Considering the patient's relapse despite treatment with antibiotics alone, it was determined to administer pneumococcal and anti-influenza vaccines at a six-month interval, excluding exacerbations.

Significant clinical and scan improvements were observed without the requirement for oral corticosteroid medication.

## Discussion

John Hunter first described mediastinal fibrosis in 1757 [1], and Nathan Oulmont followed suit in 1855 [2].

The condition affects both males and females; however, there is a slightly higher occurrence in males. It commonly occurs in individuals in their forties.

After ruling out certain causes, like long-term fungal or tuberculosis infections, as well as non-infectious, non-communicable ones like autoimmune diseases, sarcoidosis, silicosis, radiation therapy, and some medications (methysergide, practolol) [3], it is usually idiopathic.

The mediastinal fibrosis in our patient was determined to be of primitive character due to the absence of any identifiable cause throughout the etiological inquiry, especially since the patient had no history of pulmonary tuberculosis. Idiopathic fibrous mediastinitis is characterized by a prolonged period of clinical delay.

The clinical symptoms indicate the compression of the organs in the mediastinum. Their characteristics differ based on the specific organs that are impacted. These conditions may encompass pulmonary arterial hypertension, dysphonia, dysphagia, dyspnea, or superior vena cava syndrome. The latter, which is present in 60% of cases, frequently serves as an indication of the condition [4].

Imaging enables a diagnostic approach. A chest CT scan reveals a mass of variable size and density in the external area, frequently with calcifications. The MRI also enables the assessment of the extent of involvement of the mediastinal organs [5].

The appearance of the tracheobronchial tree during fiberoptic bronchoscopy might vary. It can range from inflammation of the mucosa to external compression or, more commonly, a narrowing of the tracheobronchial passage with an excessive amount of congestion and mucus [6]. The definitive diagnosis relies on the anatomopathological examination of the biopsy specimen, obtained through either imaging-guided or surgical biopsies. This examination confirms the diagnosis by revealing the presence of collagen fibrosis without necrosis and vascular abnormalities while ruling out other conditions that may mimic mediastinal fibrosis, such as hematological disorders, amyloidosis, or fibrous pseudotumor [7].

Corticosteroid therapy is the primary treatment for idiopathic mediastinal fibrosis. While it appears advantageous, there is currently no standardized protocol [8,9]. Aside from the insertion of an arterial or venous prosthesis, surgery does not seem to provide any significant advantage [10].

The degree of compression that the mediastinal organs experience primarily determines the prognosis. It is typically unfavorable. The use of corticosteroid medication has been shown to either cause regression or stabilize the condition [11,12].

## Conclusions

In conclusion, this article's examination of idiopathic mediastinal fibrosis has shed light on the nuances pertaining to this rare malady. Our careful analysis of a case study, enhanced by results from CT and bronchoscopic imaging, has shed light on the difficulties in diagnosing this condition and the therapeutic factors that must be taken into account while treating it.

By using a multidisciplinary approach, we have highlighted the importance of pulmonologists, radiologists, and thoracic surgeons working together to navigate the complexities of diagnosing and treating idiopathic mediastinal fibrosis.

Although most often idiopathic, an etiological investigation is necessary before making a diagnosis, especially of tuberculosis or an antecedent of tuberculosis, especially in an endemic country.

Our ultimate goal in writing this paper is to increase physician awareness and comprehension of idiopathic mediastinal fibrosis by adding to the amount of knowledge already available on the subject. We anticipate that our discoveries will stimulate additional investigation and ingenuity in this domain, ultimately resulting in enhanced patient results.
